# Experimental Modal Analysis and Characterization of Additively Manufactured Polymers

**DOI:** 10.3390/polym14102071

**Published:** 2022-05-19

**Authors:** Hieu Tri Nguyen, Kelly Crittenden, Leland Weiss, Hamzeh Bardaweel

**Affiliations:** 1 Institute for Micromanufacturing, College of Engineering and Science, Louisiana Tech University, Ruston, LA 71272, USA; htn005@latech.edu (H.T.N.); lweiss@latech.edu (L.W.); 2 Department of Mechanical Engineering, College of Engineering and Science, Louisiana Tech University, Ruston, LA 71272, USA; kellyc@latech.edu

**Keywords:** 3D printed materials, 3D printed polymers, characterization of 3D printed structures, ABS, fused deposition modeling, experimental modal analysis

## Abstract

Modern 3D printed components are finding applications in dynamic structures. These structures are often subject to dynamic loadings. To date, research has mostly focused on investigating the mechanical properties of these 3D printed structures with minimum attention paid to their modal analysis. This work is focused on performing experimental modal analysis of 3D printed structures. The results show that the adhesion type has the most significant impact on the vibration response and parameters obtained from the modal analysis. The average dynamic modulus, natural frequency, and damping coefficient increased by approximately 12.5%, 5.5%, and 36%, respectively, for the specimens printed using skirt adhesion compared to those printed using raft adhesion. SEM analysis suggests that the 3D printed specimens with skirt adhesion yielded flattened layers, while raft adhesion resulted in rounded layers. The flattened layers of the specimens with skirt adhesion are likely an indication of an enhanced heat transfer between the 3D printer bed and the specimen. The printed specimens with skirt adhesion are in direct contact with the printer bed during the printing process. This enhances the heat transfer between the specimen and the printer bed, causing the layers to flatten out. The enhanced heat transfer yields a better inter-layer diffusion, resulting in improved physical bonding at the layers’ interface. The improved bonding yields higher stiffnesses and natural frequencies. For the specimens with skirt adhesion, the improved heat transfer process is also likely responsible for the enhanced damping properties. The strengthened inter-layer bonding at the layer–layer interface provides better energy dissipation along the contact lines between the layers.

## 1. Introduction

Over the past two decades, additive manufacturing (AM) has progressively gained popularity in the industrial and academic fields [1,2]. AM, also known as 3D printing, offers unique advantages compared to traditional manufacturing including its ability to implement complex designs and geometries, relatively short design-to-manufacturing time, and moderately low prototyping costs, alongside its reduced residual material [1,2,3,4]. Due to its unique features and versatility, AM has several potential applications in the fields of biomechanics [5], sensors [6,7,8], and electronics [5,9], automotives [10], aerospace [11,12], defense [13], and mechanical and electromechanical systems [14].

Chief among AM technologies is fused deposition modeling (FDM) [4,15]. Currently, FDM is considered one of the most popular and user-friendly AM technologies [15]. This is mainly due to its affordability, accessibility, and adaptability with a wide range of commercially available thermoplastic filaments and materials including ABS (acrylonitrile butadiene styrene) and PLA (polylactic acid). FDM 3D printers extrude the thermoplastic filaments which then pass through heated nozzles. By melting these thermoplastics, an FDM 3D printer builds the desired structure by layering the melted filament [16]. However, the layer-by-layer printing process in FDM can lead to multiple challenges including warpage and shrinking, poor adhesion between layers, and rough, or poor, surface quality finishing [12,17,18,19]. Consequently, the properties of structures printed using FDM are often anisotropic due to material layering during the 3D printing process [20].

To date, several studies have investigated the effects of different 3D printing parameters on the mechanical [21] and thermal [22] properties of 3D printed parts. For example, a number of studies have investigated the effects of the raster orientation and angle on the mechanical properties of 3D printed specimens including the tensile strength [23,24,25,26], fatigue [27], fracture [12], and flexural and impact strengths [23,25]. Moreover, the effects of other major parameters on the mechanical properties of 3D printed specimens such as the printing orientation [28,29,30], layer thickness [19,30,31,32], and nozzle temperature [33,34] and diameter [35], along with the printing speed [36,37], have also been investigated. For instance, Chacon et al. [29] studied the effect of three main printing parameters, i.e., printing orientation, layer thickness, and feed rate, on the mechanical properties of PLA specimens produced using FDM 3D printing. The results from this study showed that the upright orientation exhibited the lowest mechanical properties, while the on-edge and flat orientations resulted in the highest mechanical strength. The results also revealed that both the feed rate and layer thickness have inverse relationships with ductility.

In another recent study, Rodríguez-Panes et al. [38] presented a comparative study focused on comparing the tensile mechanical behavior of PLA and ABS specimens produced using an FDM 3D printer. In this study, the effects of the infill density, layer thickness, and printing orientation on the mechanical properties of 3D printed specimens were investigated. The results from this study showed that there was less variation in the mechanical properties of ABS printed specimens compared to PLA specimens. The infill density showed the greatest effect on the mechanical properties of the 3D printed specimens. In another reported study [39], ABS and polycarbonate (PC) specimens were 3D printed, and the effects of the raster orientation and printing orientation on the directional properties of the specimens were investigated. The study concluded that both the raster and print orientation had insignificant effects on the Poisson ratio and the Young’s modulus of elasticity of the 3D printed ABS specimens. Meanwhile, both shear properties, i.e., modulus of elasticity and shear yield strength, varied significantly, i.e., up to 33%, in ABS specimens. Examples of other studies that have investigated the mechanical properties of 3D printed specimens using FDM technology include those reported here [40,41,42,43,44,45]. Additionally, comprehensive articles have recently reviewed the subject of the mechanical characterization of 3D printed structures [46,47] as well as the effect of various printing parameters on their mechanical properties [48,49,50].

In this work, an experimental effort that focuses on the evaluation of the modal analysis and vibration response, as well as the characterization, of FDM additively manufactured structures is presented. This work also provides insight into the effects of 3D printing parameters on these structures. Parameters included in this study were layer thickness, adhesion type, and printing direction.

The aforementioned literature describes recent efforts focused on studying the mechanical properties of structures fabricated using FDM-based 3D printing technologies. While there has been significant effort in the evaluation of material properties, there remains a deficit of modal and vibration analysis in the manufactured structures themselves. To date, the effects of various printing parameters on the modal and vibration response and characterization of these 3D printed structures have been rarely investigated [51]. Nonetheless, these additively manufactured structures are gaining popularity in dynamic applications which are often subject to external vibrations and dynamic loading. Examples of these externally excited vibration structures include flexing electronics, sensors, actuators, and robotics. Therefore, vibration modal analysis becomes increasingly necessary to ensure the dynamic reliability and structural integrity of 3D printed components.

The structure of this article is organized as follows: Section 2 discusses manufacturing processes and materials used in this work. Experimental methods and techniques are detailed in Section 3. The results and findings from modal analysis and mechanical characterization tests are detailed in Section 4. The discussion of the results is presented in Section 5. The summary and final remarks from the current work are found in Section 6.

## 2. Materials and Fabrication

The thermoplastic material used in this study was ABS. While the modal analysis presented in this work can be extended to a variety of 3D printing filaments and composites, in this study, ABS was selected as the primary 3D printing material due to its unique properties including its robustness, rigidity, chemical stability, and durability [25]. Additionally, ABS represents a type of thermoplastic that is gaining more popularity in many industries as noted previously. In automotive applications, for example, it is used to manufacture instrument panels, seating, interior and exterior trims, lighting, mud flaps, bumpers, and fenders [52,53].

**Figure 1:**
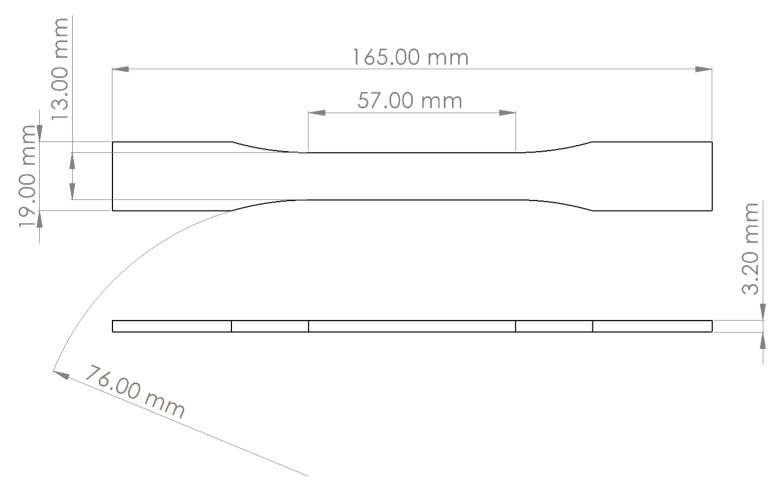
Design, geometry, and dimensions of the 3D printed specimens used for baseline mechanical characterization following the ASTM D638 02a standards.

In this work, fabrication of the ABS specimens started by drying ABS pellets at 80 °C for approximately 5 h using a Vulcan oven (Vulcan 3-550). The dried pellets were then fed into a Filabot extruder (Filabot Ex2) at 175 °C. The extruded filament was collected by a spooler system to maintain the filament’s diameter at approximately 2.85 mm. Next, the extruded filament was fed into a Lulzbot Mini 3D desktop printer to print the desired specimens for mechanical and vibration testing and characterization. The geometries and dimensions of the 3D printed specimens used for mechanical characterization are shown in Figure 1. These specimens followed specifications described by the ASTM D638-02a standards (type I).

**Figure 2:**
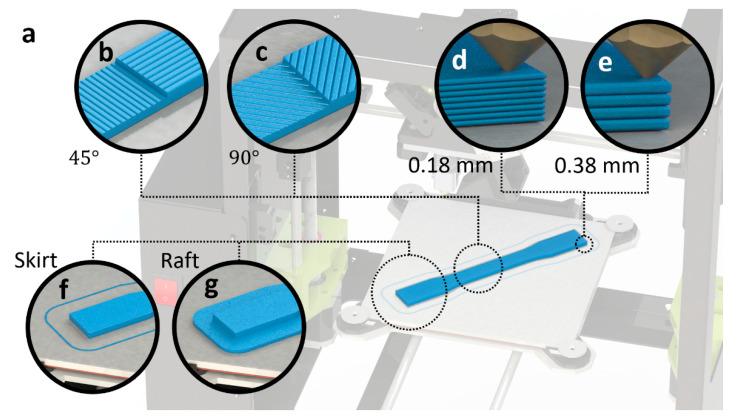
An illustrative drawing of the main 3D printing parameters varied and studied in this work including the printing direction, layer thickness, and adhesion type: (**a**) a sample on the print bed; (**b**,**c**) a comparison between two printing directions, 45° and 90°, respectively; (**d**,**e**) a comparison between two layer thicknesses, 0.18 mm and 0.38 mm, respectively; and (**f**,**g**) a comparison between the skirt and raft adhesion types, respectively.

Table 1 lists the main parameters that were used during the 3D printing process including the infill density, temperatures, and print speed. Specific to this study, the layer thickness, adhesion type, and printing direction parameters were varied during the 3D printing process to study the effects of these fabrication settings on the modal analysis and free-vibration response of the printed parts. These three parameters are described in Figure 2. Specimens with layer thicknesses of 0.18 mm and 0.38 mm, skirt and raft adhesion types, and printing directions of 45° and 90° were prepared. This resulted in eight types of 3D printed specimens, as shown in Table 2. Following the ASTM recommendations, five samples of each type of ABS specimen were fabricated and tested in this work.

## 3. Experimental Apparatus

**Figure 3:**
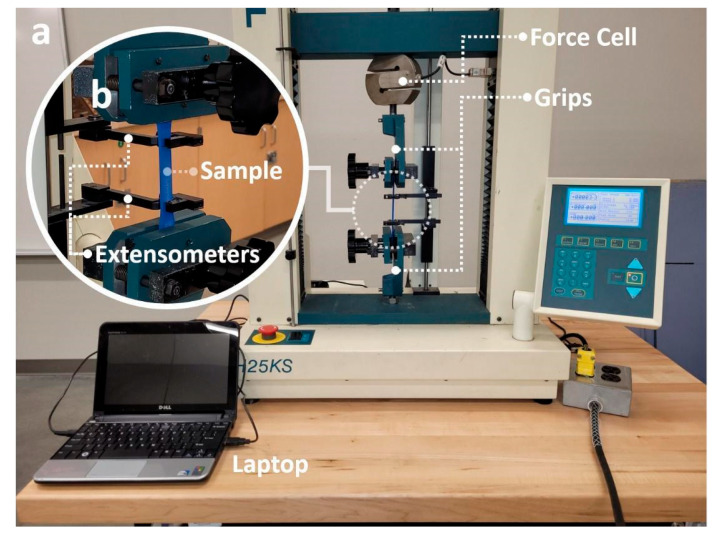
Mechanical characterization setup used for testing the 3D printed specimens: (**a**) apparatus used to perform the tensile tests, and (**b**) a close-up picture of the loaded specimen during the tests.

Mechanical characterization of the 3D printed specimens was performed using the experimental setup shown in Figure 3. Uniaxial tensile tests were performed using a Tinus Olsen machine (H25KS). The specimen was loaded into the machine as shown in Figure 3 and then strained by a 5 kN load cell at a 5 mm/min rate. The strain rate was selected to be within the specified ASTM standards. The displacement of the loaded specimen was measured using an axial extensometer. Both the tensile force and displacement were recorded on a laptop for later analysis, as shown in Figure 3. Following the techniques outlined in the ASTM D638 02a testing standards, the recorded force and displacement were used to calculate the stress and strain as well as other mechanical properties including Young’s modulus, ultimate tensile strength, yield strength, and maximum strain. The methods and equations used to calculate these mechanical properties are described in Appendix A.

**Figure 4:**
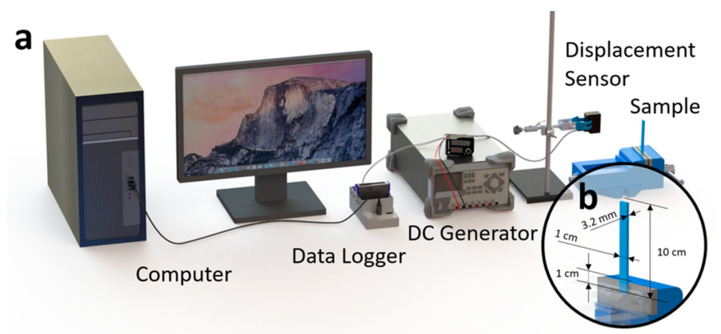
Apparatus used for experimental model analysis of 3D printed specimens: (**a**) an illustrative figure showing the connection and configuration of the experiment setup and (**b**) the design and configuration of the 3D printed cantilever and its clamping conditions.

In addition to the mechanical characterization baseline, experimental modal analysis was performed using the apparatus depicted in Figure 4. For these tests, cantilever-shaped structures were 3D printed and used to perform the experimental modal analysis. Figure 4 depicts the design and geometry of the 3D printed cantilever as well as the clamping conditions. The experimental setup shown in Figure 4 consists of a vice, a displacement sensor (Keyence IL-100), a DC generator (Rigol DP832), a data logger (NI9205), and a PC. The cantilever was secured in position using a vice, as shown in Figure 4. The displacement sensor was powered by the DC generator and used to track the displacement of the cantilever tip as it went through free vibration. The collected time-series data were then sent to the data logger and, finally, the PC where the data were stored and analyzed. Time-series data collected from this experiment were used to perform modal analysis and frequency-domain analysis in order to extract useful modal parameters including the resonant frequency, $f_{n}$, dynamic modulus, $E_{d}$, and damping coefficient, ζ. Appendix A contains the details and formulas used for modal analysis.

In addition to these macroscopic characterization tests, scanning electron microscope (SEM) analysis was carried out in order to investigate the microscopic structures of the 3D printed ABS specimens. Broken specimens from the tensile tests were prepared by coating the fractured surface with a 3 nm layer of gold in order to perform the SEM imaging. A Hitachi S-4800 was operated at 3 kV and 35x magnification to yield images that were used to gain insight into the overall macroscopic measured properties and behaviors of the ABS specimens.

## 4. Results

**Figure 5:**
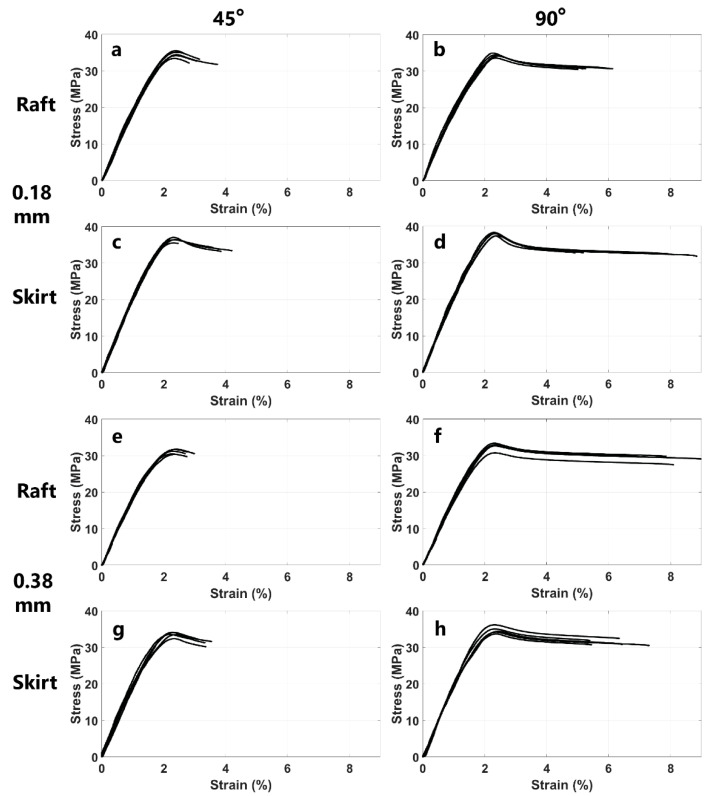
Measured stress–strain curves of each group of examined specimens. Five specimens of each type were tested: (**a**) R-45-0.18 mm, (**b**) R-90-0.18 mm, (**c**) S-45-0.18 mm, (**d**) S-90-0.18 mm, (**e**) R-45-0.38 mm, (**f**) R-90-0.38 mm, (**g**) S-45-0.38 mm, and (**h**) S-90-0.38 mm.

Figure 5 shows the stress–strain curves of the 3D printed ABS specimens obtained using the experiment apparatus shown in Figure 3. The tensile stress–strain data were measured for five specimens of each type shown in Table 2. The collected data from these experiments were used to calculate the main mechanical properties of the 3D printed specimens including the ultimate strength, yield strength, Young’s modulus of elasticity, maximum strain, and strain energy density. These mechanical properties are summarized in Table 3. The results shown in Figure 5 exhibit high repeatability and very low deviation across the data collected from the five specimens. Additionally, Figure 5 suggests that the 3D printed specimens with a 90-degree printing direction exhibited a higher maximum strain and, consequently, a larger strain energy density compared to the 3D printed specimens with a 45-degree printing direction. This is likely because, during the tensile test, those 45-degree specimens were pulled in a transverse direction with respect to the 3D printed layers.

The results shown in Table 3 suggest that the adhesion type, i.e., skirt versus draft, had a major impact on the mechanical properties of the 3D printed specimens. For the fixed layer thicknesses and printing directions, the specimens printed with skirt adhesion exhibited a higher ultimate strength, yield strength, and modulus of elasticity. For instance, for samples printed with a 0.18 mm thickness and a 45-degree printing direction, the specimens with skirt adhesion (S-45-0.18 mm) measured an ultimate strength, yield strength, and Young’s modulus of 36.40 MPa, 31.42 MPa, and 2100 MPa, respectively, compared to 34.55 MPa, 29.51 MPa, and 1970 MPa measured for the raft-adhesion-type specimens with the same thickness and printing direction (R-45-0.18 mm).

In addition, the results in Table 3 suggest that varying the printing direction while fixing the other two parameters, i.e., the layer thickness and adhesion type, affected some of the mechanical properties of the 3D printed specimens. For instance, compared to the 45-degree printing direction, the 90-degree-printed specimens had a higher maximum strain and strain energy density. As shown in Table 3, the specimens with a 0.38 mm layer thickness and the skirt adhesion type experienced a maximum strain of 6.199% and a strain energy density of 1.738 MPa compared to the maximum strain of 3.264% and strain energy density of 0.781 MPa when the printing direction was changed from 90 degrees to 45 degrees, i.e., S-90-0.38 mm and S-45-0.38 mm, respectively. Moreover, the results shown in Table 3 reveal that the combination of skirt adhesion and the 90-degree printing direction resulted in an overall improvement in the mechanical properties of the 3D printed specimens. For instance, comparing the first and fourth rows in Table 3, one can notice that for the fixed layer thickness of 0.18 mm, the combination of skirt adhesion and the 90-degree printing direction, i.e., S-90-0.18 mm, yielded superior mechanical properties compared to the specimens with a similar layer thickness, i.e., 0.18 mm, with raft adhesion and the 45-degree printing direction, i.e., R-45-0.18 mm. Here, S-90-0.18 mm specimens exhibit approximately 10%, 10%, 10%, 120%, and 180% improvement in the ultimate strength, yield strength, Young’s modulus, maximum strain, and strain energy density, respectively, when compared to the specimens with a similar layer thickness, i.e., 0.18 mm, with the raft adhesion type and the 45-degree printing direction, i.e., R-45-0.18 mm.

**Figure 6:**
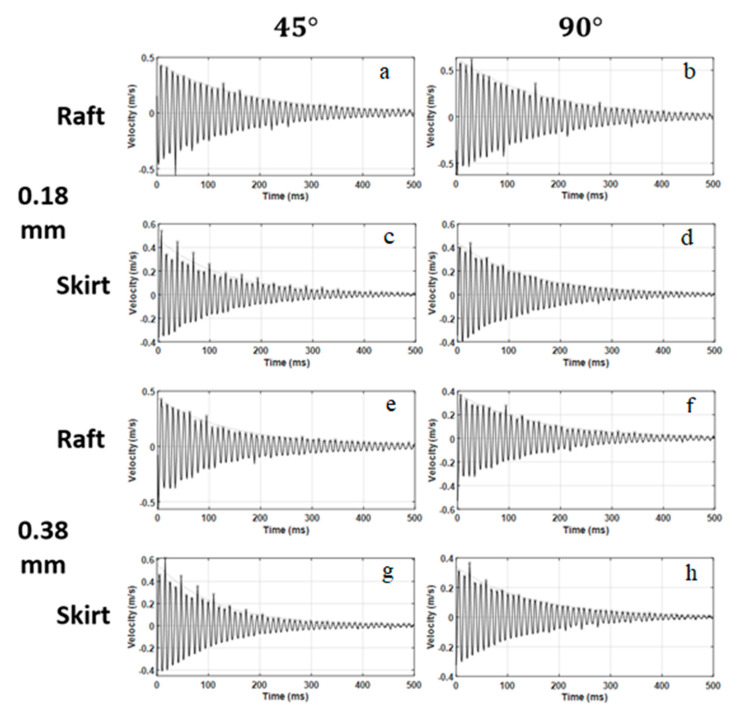
Time-series ring-down free-vibration response obtained from the tested 3D printed cantilevers: (**a**) R-45-0.18 mm, (**b**) R-90-0.18 mm, (**c**) S-45-0.18 mm, (**d**) S-90-0.18 mm, (**e**) R-45-0.38 mm, (**f**) R-90-0.38 mm, (**g**) S-45-0.38 mm, and (**h**) S-90-0.38 mm.

**Figure 7:**
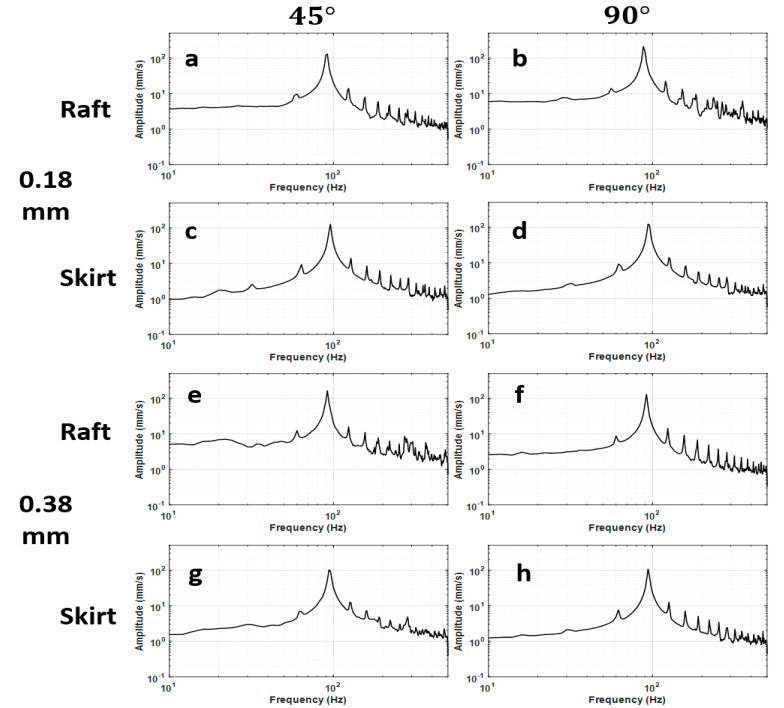
Frequency-domain response of the 3D printed cantilevers: (**a**) R-45-0.18 mm, (**b**) R-90-0.18 mm, (**c**) S-45-0.18 mm, (**d**) S-90-0.18 mm, (**e**) R-45-0.38 mm, (**f**) R-90-0.38 mm, (**g**) S-45-0.38 mm, and (**h**) S-90-0.38 mm.

**Figure 8:**
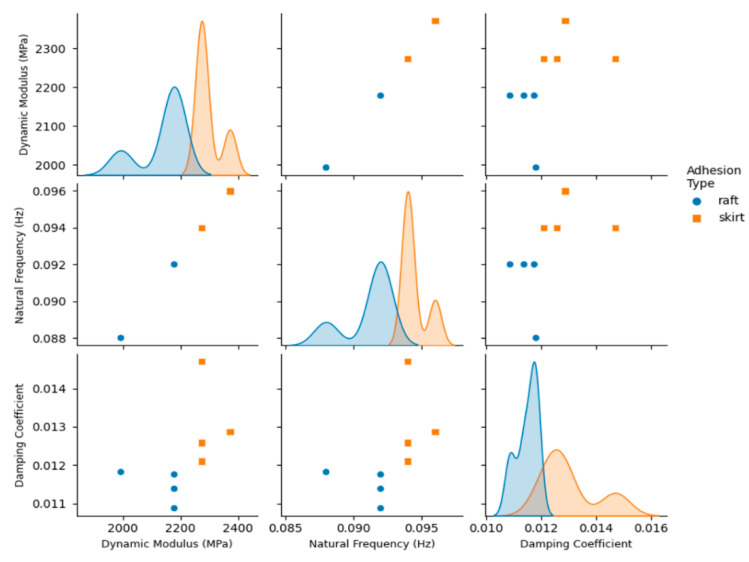
Pair plots of the modal analysis parameters obtained from the free-vibration tests of the 3D printed cantilevers. The variable of interest is the adhesion type (skirt and raft).

**Figure 9:**
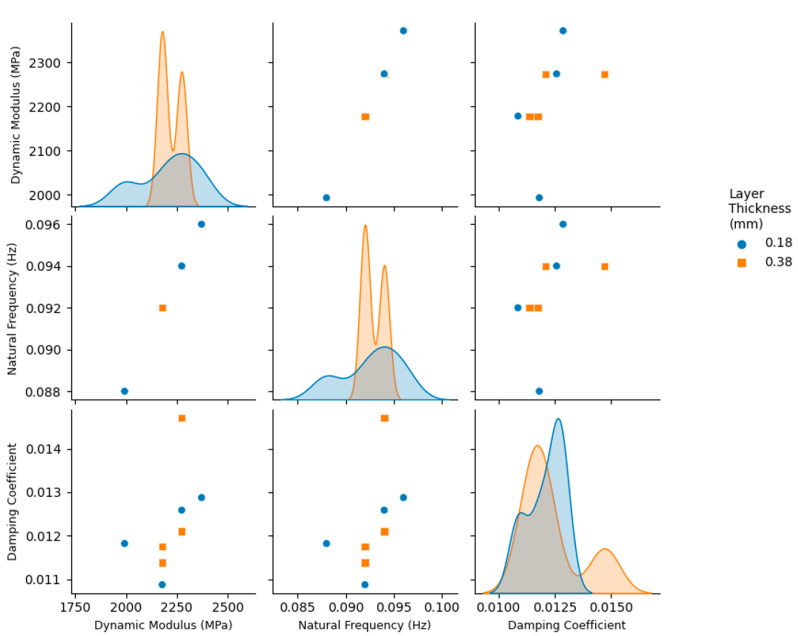
Pair plots of the modal analysis parameters obtained from the free-vibration tests of the 3D printed cantilevers. The variable of interest is the layer thickness (0.18 mm and 0.38 mm).

**Figure 10:**
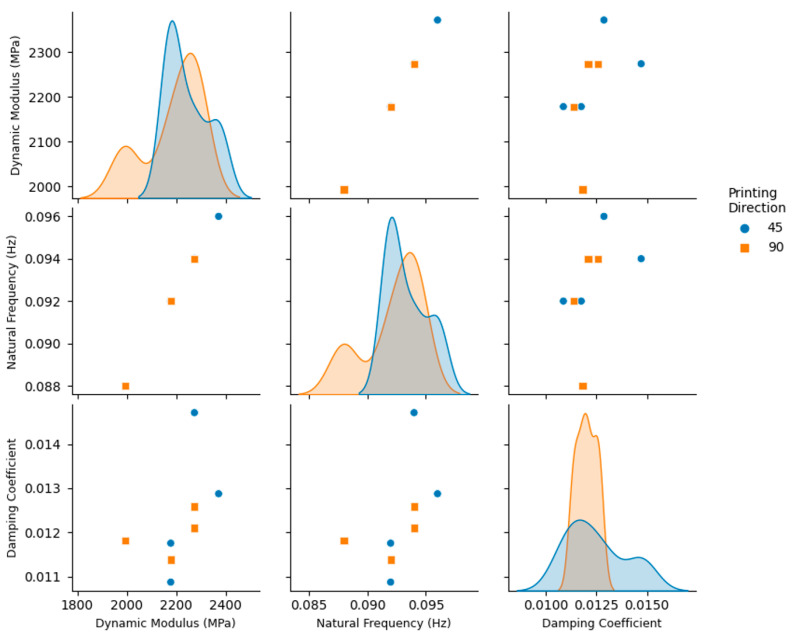
Pair plots of the modal analysis parameters obtained from the free-vibration tests of the 3D printed cantilevers. The variable of interest is the printing direction (45 and 90 degrees).

The vibration response of the 3D printed ABS cantilevers is examined in Figure 6, Figure 7, Figure 8, Figure 9 and Figure 10. The time-series free-vibration and frequency responses shown in Figure 6 and Figure 7, respectively, were obtained using the vibration characterization experiment setup shown in Figure 4. In these experiments, the eight groups of the 3D printed cantilevers, shown in Table 2, were fixed on the experimental setup, and the time-series free-vibration and frequency responses of the specimens were measured. Five samples of each type were tested. Moreover, Figure 8, Figure 9 and Figure 10 display the main parameters obtained from the experimental modal analysis for the 3D printed ABS cantilevers including the natural frequency, $f_{n}$, damping coefficient, ζ, and dynamic modulus, $E_{d}$. Details of the modal analysis technique and formulas used to analyze the experimental data are discussed in Appendix A.

The results shown in Figure 8, Figure 9 and Figure 10 reveal that, between all three parameters investigated in this study, i.e., adhesion type, printing direction, and layer thickness, the adhesion type has the most significant effect on the vibration response and parameters obtained from the modal analysis. As shown in Figure 8, 3D printed cantilevers with the skirt adhesion type exhibited higher dynamic moduli, natural frequencies, and damping coefficients compared to the cantilevers printed with raft adhesion. As visible in Figure 8, any layer thickness and print direction yielded an increased average dynamic modulus, natural frequency, and damping coefficient. These increased from 2.09 GPa, 90 Hz, and 0.011 to 2.35 GPa, 95 Hz, and 0.015 as the adhesion type was modified from raft to skirt. This corresponds to an approximately 12.5%, 5.5%, and 36% increase in the Young’s modulus, natural frequency, and damping coefficient, respectively, of the cantilevers printed using skirt adhesion compared to those printed using raft adhesion. Lastly, Figure 9 and Figure 10 suggest that neither the layer thickness nor the printing direction had a major impact on the measured dynamic parameters.

**Figure 11:**
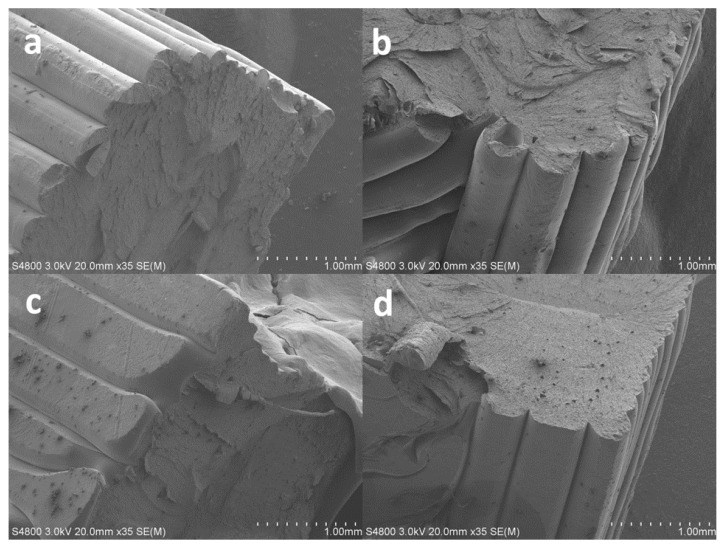
SEM images of the broken 3D printed specimens with a 0.18 mm layer thickness: (**a**) R-45-0.18 mm, (**b**) R-90-0.18 mm, (**c**) S-45-0.18 mm, and (**d**) S-90-0.18 mm.

**Figure 12:**
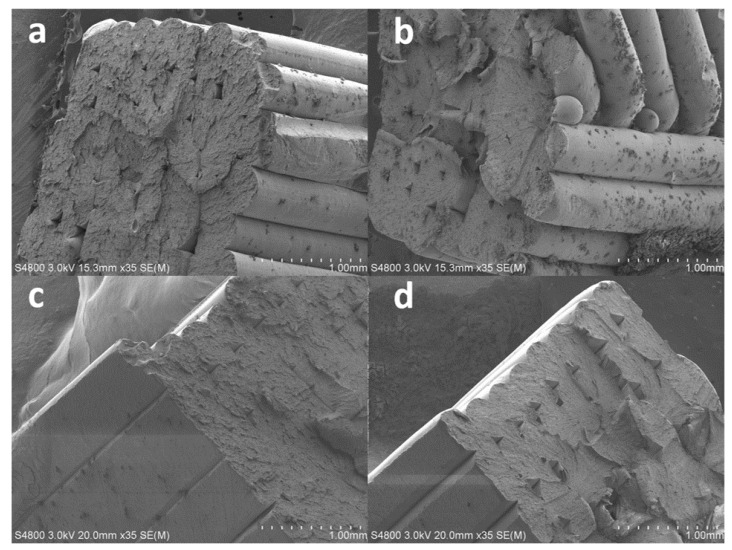
SEM images of the broken 3D printed specimens with a 0.38 mm layer thickness: (**a**) R-45-0.38 mm, (**b**) R-90-0.38 mm, (**c**) S-45-0.38 mm, and (**d**) S-90-0.38 mm.

The results from the SEM analysis of the 3D printed specimens are shown in Figure 11 and Figure 12. The results show cross-sectional areas of the eight types of 3D printed specimens shown in Table 2. The layer-by-layer printing and the two printing directions, i.e., 45^o^ and 90°, are evident in these SEM images. SEM analysis revealed the effect of the adhesion type, i.e., skirt versus raft, on the 3D printed layer. As shown in Figure 11 and Figure 12, regardless of the printing direction and layer thickness, 3D printed specimens with raft adhesion showed a rounded (almost circular) cross-sectional area of the layers. However, 3D printed specimens with skirt adhesion exhibit flattened layers with an almost rectangular cross-sectional area. A key contributor to this difference is the fact that the 3D printed specimens with skirt adhesion were in direct contact with the 3D printer bed during the printing process which is elevated to a temperature of 110 °C. This was not the case for the 3D printed specimens with raft adhesion. Direct contact enhances the heat transfer process between the 3D printed specimen and the 3D printer bed, causing the layer to better spread, i.e., flatten, before solidification and attainment of the final, flattened shape.

## 5. Discussion

The results from the characterization tests exhibit a very small standard deviation between the group of five tested specimens of each 3D printed type shown in Table 2. Most notably, the results indicate that the adhesion type, i.e., raft versus skirt, had the most significant impact on the vibration response and modal analysis. Overall, the specimens printed with the skirt adhesion type showed a superior vibration response and improved modal analysis parameters. These skirt-adhesion-type specimens exhibited a higher natural frequency, a higher dynamic modulus, and improved damping properties. Moreover, the use of skirt adhesion during the 3D printing process improved some of the mechanical properties and resulted in a higher ultimate strength, yield strength, and modulus of elasticity.

This superior performance of the skirt-adhesion-type specimens is likely due to the improved and uniform heat transfer between the 3D printer bed and the specimens during the 3D printing process. As suggested by the SEM analysis, skirt adhesion yielded flattened layers, while raft adhesion resulted in rounded layers. The flattened layers observed in the SEM images of the specimens with skirt adhesion are likely an indication of an enhanced heat transfer process between the 3D printer bed and the specimen during the 3D printing process. Presumably, this improved heat transfer results in better diffusion between the layers during the 3D printing process, therefore enhancing the bonding at the inter-layer interface. Consequently, this enhancement results in a higher mechanical strength, modulus of elasticity, and dynamic modulus. The improved moduli yielded an increase in stiffness and, therefore, higher natural frequencies. Similarly, for the specimens with skirt adhesion, the improved heat transfer process between the 3D printer bed and the specimens is likely responsible for the enhancement in the damping properties. That is, the improved inter-layer bonding at the layer–layer interface provides better energy dissipation along the contact lines between the 3D printed layers. Consequently, this enhanced energy dissipation yields higher damping coefficients and improves the overall damping properties of the 3D printed specimens, as evident in the results shown in Figure 8, Figure 9 and Figure 10.

Motivated by the growing popularity of FDM 3D printing as a cost-effective and user-friendly technique for manufacturing various dynamic structures, this work sheds new light on the modal analysis and vibration response of FDM 3D printed specimens. The results from this work demonstrate the effect of the three studied parameters, i.e., adhesion type, printing direction, and layer thickness, on the vibration response of the 3D printed specimens. This work offers a roadmap for the role of these 3D printing parameters in determining vital and relevant dynamic characteristics including damping, stiffness, and resonant frequency of 3D printed dynamic structures such as sensors, actuators, robotics, and flexing components. The ability to control and tune some of the dynamic characteristics and parameters obtained from the modal analysis of these structures is essential to a successful design and implementation. For instance, low stiffness values are favorable for designing force sensors in order to obtain a high sensing resolution [54]. In strain sensors, high resonant frequencies are desired [55]. Through careful consideration of 3D printing parameters, one can tune the overall dynamics of the 3D printed structures in order to obtain the desired response and performance.

## 6. Conclusions

To date, state-of-the-art efforts have primarily focused on investigating the mechanical properties of 3D printed structures as well as the effect of various printing parameters on their mechanical behavior. These additively manufactured structures are gaining more popularity in dynamic applications including flexing electronics, sensors, actuators, and robotics. These structures are often subject to dynamic loading. Therefore, vibration modal analysis becomes necessary to ensure the dynamic reliability and structural integrity of these 3D printed components.

This work focused on performing experimental modal analysis of FDM additively manufactured structures. The ABS thermoplastic was selected as the primary 3D printing material. The fabrication of the specimens as well as the experimental characterization methods is presented in this article. This work also provides insight into the effects of various 3D printing parameters on the modal analysis of these printed structures. Specimens with layer thicknesses of 0.18 mm and 0.38 mm, skirt and raft adhesion types, and printing directions of 45° and 90° were manufactured and tested.

The results suggest that the adhesion type has the most significant impact on the vibration response and results from the modal analysis. The skirt-adhesion-type specimens exhibited a higher natural frequency, a higher dynamic modulus, and improved damping properties. The results show that, for any layer thickness and printing direction, the average dynamic modulus, natural frequency, and damping coefficient increased by approximately 12.5%, 5.5%, and 36%, respectively, for the specimens printed using skirt adhesion compared to those printed using raft adhesion. Moreover, the use of skirt adhesion during the 3D printing process resulted in a higher ultimate strength, yield strength, and modulus of elasticity.

The enhanced performance of the skirt-adhesion-type specimens was likely due to the enhanced heat transfer between the 3D printer bed and the specimen during the 3D printing process. The results from the SEM analysis reveal that, regardless of the layer thickness and printing direction, specimens with skirt adhesion yielded flattened layers, while specimens with raft adhesion yielded rounded layers. The flattened layers observed in the SEM analysis of the specimens with skirt adhesion are an indication of an enhanced heat transfer process between the 3D printer bed and the specimen during the 3D printing process. This is because, for skirt adhesion, the 3D printed specimens are in direct contact with the 3D printer bed. This direct contact enhances the heat transfer process between the 3D printed specimen and the 3D printer bed, causing the layer to flatten out as revealed by the SEM images. Presumably, the enhanced heat transfer yielded a better inter-layer diffusion, resulting in improved physical bonding at the inter-layer interface. Thus, this enhanced bonding between layers resulted in a higher mechanical strength, modulus of elasticity, and dynamic modulus. Consequently, the improved moduli yielded a higher stiffness which led to higher natural frequencies. For the specimens with skirt adhesion, the improved heat transfer process between the 3D printer bed and the specimens was responsible for the enhanced damping properties. The strengthened inter-layer bonding at the layer–layer interface provided better energy dissipation along the contact lines between the layers. This enhanced energy dissipation yielded higher damping coefficients and improved the overall damping properties of the 3D printed specimens.

## Figures and Tables

**Table 1:** Settings and parameters of 3D printer used to produce the specimens.

Specs and Settings	Value
Layer Height	0.18 mm or 0.38 mm
Top/Bottom Line Direction	[0, 90] or [45, 135]
Infill Density	99.99%
Infill Line Directions	[0, 90] or [45, 135]
Default Printing Temperature	240 °C
Build Plate Temperature	110 °C
Print Speed	60 mm/s
Build Plate Adhesion Type	Skirt or Raft

**Table 2:** A summary of the different types of 3D printed specimens considered in this study and their corresponding parameters.

Label	Adhesion Type	Printing Direction	Layer Thickness
**R-45-0.18 mm**	Raft	45°	0.18 mm
**R-45-0.38 mm**	Raft	45°	0.38 mm
**R-90-0.18 mm**	Raft	90°	0.18 mm
**R-90-0.38 mm**	Raft	90°	0.38 mm
**S-45-0.18 mm**	Skirt	45°	0.18 mm
**S-45-0.38 mm**	Skirt	45°	0.38 mm
**S-90-0.18 mm**	Skirt	90°	0.18 mm
**S-90-0.38 mm**	Skirt	90°	0.38 mm

**Table 3:** Measured mechanical properties of the 3D printed specimens studied in this work. M: mean; SD: standard deviation.

	$\mathrm{Ultimate}\;\mathrm{Strength},\;\sigma_{u}(\mathrm{MPa})$		$\mathrm{Yield}\;\mathrm{Strength},\;\sigma_{y}(\mathrm{MPa})$		Young Modulus, E		$\mathrm{Maximum}\;\mathrm{Strain},\;\varepsilon_{max}(\%)$		Strain Energy Density, SED(Mpa)	
	**M**	**SD**	**M**	**SD**	**M**	**SD**	**M**	**SD**	**M**	**SD**
**R-45-0.18 mm**	34.55	0.83	29.51	1.66	1970	102	3.099	0.408	0.729	0.125
**R-90-0.18 mm**	34.30	0.50	24.99	2.50	2085	153	5.525	0.513	1.488	0.177
**S-45-0.18 mm**	36.40	0.55	31.42	3.03	2100	131	3.267	0.882	0.831	0.306
**S-90-0.18 mm**	37.87	0.45	31.97	1.756	2155	85	6.814	1.736	2.034	0.585
**R-45-0.38 mm**	31.10	0.67	25.60	1.38	1949	84	2.748	0.173	0.573	0.054
**R-90-0.38 mm**	32.54	1.03	29.39	0.25	1836	114	7.830	0.838	2.111	0.240
**S-45-0.38 mm**	33.54	0.70	28.57	1.54	2035	163	3.264	0.240	0.781	0.070
**S-90-0.38 mm**	34.71	0.96	30.05	2.39	2021	57	6.199	0.797	1.738	0.254

**Figure A1:**
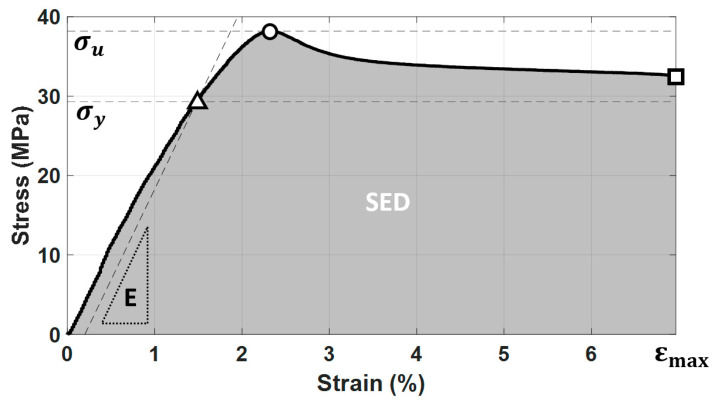
A representative stress–strain curve obtained from the tensile tests which were performed on the specimens reported in this work.

**Figure A2:**
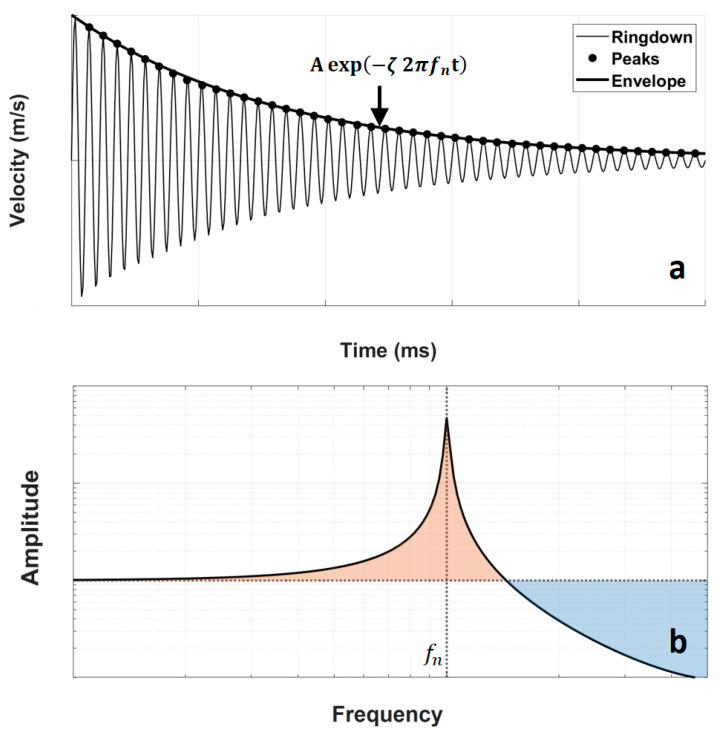
A representative vibration response from the cantilever specimen showing: (**a**) the ringdown plot with its envelope curve and (**b**) the frequency-domain plot of the specimen.

## Data Availability

Data are contained within the article.

## Appendix A

Mechanical properties from the tensile test were calculated using the following equations [56]:

Ultimate stress:

(A1) $$\sigma_{u}=Max\left(\sigma \right)$$

Maximum strain:

(A2) $$\varepsilon_{max}=Max\left(\varepsilon \right)$$

Strain energy density:

(A3) $$SED=\int_{0}^{\varepsilon_{max}}\sigma \;d\varepsilon$$

These mechanical properties are illustrated graphically in Figure A1.

The dynamic modulus was calculated as follows [57,58]:

(A4) $$E_{d}=\frac{{\left(2\pi f_{n}\right)}^{2}\;0.09^{4}\rho }{3.516^{2}\;\left(\frac{bh^{3}}{12}\right)}$$

In (A4), ρ is the density of the cantilever, and b and h are the width and thickness, respectively, of the cross-section of the cantilever.

Figure A2 shows an illustration of the free-vibration response [59].
